# Epidemiology and Survival Outcomes for Patients With NSCLC in Scandinavia in the Preimmunotherapy Era: A SCAN-LEAF Retrospective Analysis From the I-O Optimise Initiative

**DOI:** 10.1016/j.jtocrr.2021.100165

**Published:** 2021-03-24

**Authors:** Simon Ekman, Pia Horvat, Mats Rosenlund, Anne Mette Kejs, Dony Patel, Ariadna Juarez-Garcia, Laure Lacoin, Melinda J. Daumont, John R. Penrod, Odd Terje Brustugun, Jens Benn Sørensen

**Affiliations:** a Thoracic Oncology Center, Department of Oncology-Pathology, Karolinska University Hospital, Karolinska Institutet, Stockholm, Sweden; b Real-World Evidence Solutions, IQVIA, London, United Kingdom; c Real-World & Analytics Solutions, IQVIA, Solna, Sweden; d Department of Learning, Informatics, Management and Ethics (LIME), Karolinska Institutet, Stockholm, Sweden; e Real-World & Analytics Solutions, IQVIA, Copenhagen, Denmark; f Worldwide Health Economics & Outcomes Research, Bristol Myers Squibb, Uxbridge, United Kingdom; g Epi-Fit, Bordeaux, France; h Worldwide Health Economics & Outcomes Research, Bristol Myers Squibb, Braine-L’Alleud, Belgium; i Worldwide Health Economics & Outcomes Research, Bristol Myers Squibb, Princeton, New Jersey; j Section of Oncology, Drammen Hospital, Vestre Viken Hospital Trust, Drammen, Norway; k Department of Oncology, Rigshospitalet, Copenhagen, Denmark

**Keywords:** Epidemiology, Non–small cell lung cancer, Survival, I-O Optimise, Real-world data

## Abstract

**Introduction:**

SCAN-LEAF, part of the I-O Optimise initiative, is a retrospective, longitudinal study investigating the epidemiology, clinical care, and outcomes for patients with NSCLC in Scandinavia. We report overall survival (OS) trends for patients diagnosed with NSCLC in Sweden and Denmark between 2005 and 2015.

**Methods:**

Swedish and Danish cohorts were established by linking national registries. Data on all adults diagnosed with incident NSCLC from January 1, 2005, to December 31, 2015, were included. For temporal analyses of OS trends, patients were stratified by TNM stage and histology.

**Results:**

Between 2005 and 2015, a total of 30,067 and 31,939 patients from Sweden and Denmark, respectively, were diagnosed with NSCLC; the most common histological subtype was nonsquamous cell carcinoma (56.9% and 53.0%) and 48.4% and 51.6% were diagnosed at stage IV. Over the study period, significant improvements in short-term survival (1 y) were observed for patients with nonsquamous cell carcinoma in both countries, regardless of disease stage at diagnosis; however, improvements in longer-term survival (5 y) were limited to patients with stage I and II disease only. Conversely, among patients with squamous cell histology, improvements in short-term survival were only observed for stage I disease in Sweden and stage IIIA disease in Denmark, while significant improvements in longer-term survival were seen only for stage IIIA NSCLC in both countries.

**Conclusions:**

Despite some survival improvements between 2005 and 2015, an unmet need remains for patients with advanced NSCLC, particularly those with squamous cell histology. Future analyses will evaluate the impact of newer treatments on OS in NSCLC.

## Introduction

Lung cancer is the leading cause of cancer death worldwide.^1^ In 2018, there were approximately 4000 and 5000 new cases of lung cancer in Sweden and Denmark, respectively, accounting for 6.6% and 12.1% of all cancers diagnosed, and 16.1% and 26.1% of all cancer-related deaths.^2^ Around 85% of all lung cancers are NSCLC, and most of these are of the nonsquamous (NSQ) subtype.^3^^,^^4^ Currently, about two-thirds of patients with NSCLC are diagnosed with locally advanced or metastatic disease (stage III or IV) according to the TNM staging classification system.^5^^,^^6^ For patients with stage III or IV NSCLC, treatment options are limited and prognosis remains dire; 5-year survival rates for patients with metastatic NSCLC are less than 5%.^7^^,^^8^

Historically, advanced NSCLC was primarily treated with platinum-based chemotherapy in first line. Advances in the understanding of tumor biology and the identification of oncogenic drivers, such as mutations in the *EGFR* gene and rearrangements of the *ALK* gene, have led to the development of targeted therapies such as tyrosine kinase inhibitors (TKIs).^9^ TKIs were first launched for the treatment of NSCLC in Sweden and Denmark in 2010 and have subsequently improved the treatment landscape.^10^ Immunotherapy with immune checkpoint inhibitors (ICIs) has demonstrated potential to improve outcomes in patients with advanced NSCLC.^11^, ^12^, ^13^, ^14^, ^15^, ^16^ Since 2015, several ICIs have been approved in Europe for the second-line treatment of NSCLC (nivolumab, pembrolizumab, atezolizumab) and as consolidation therapy after chemoradiotherapy in locally advanced unresectable NSCLC with programmed death-ligand 1 expression greater than or equal to 1% (durvalumab).^17^, ^18^, ^19^, ^20^ More recently, since 2017, ICI treatment alone (pembrolizumab)^18^ or combined with chemotherapy (pembrolizumab, atezolizumab, nivolumab)^17^, ^18^, ^19^ has been approved for the first-line treatment of advanced or metastatic NSCLC. For patients with nonmetastatic NSCLC, ongoing clinical trials are investigating neoadjuvant, adjuvant, and perioperative use of ICIs.^21^

In this rapidly changing treatment landscape, it is important to assess the impact of newer therapies on patient survival to help inform future treatment decisions and standards of care. This requires improved understanding of NSCLC disease epidemiology and outcomes prior to their development. Particularly, a comprehensive preimmunotherapy “baseline” needs to be established to track changes in patient outcomes and survival as immunotherapies become a routine part of clinical practice. Real-world data provide valuable evidence as new treatments are introduced and the standard of care evolves; they can provide clinical insights, complementing data from randomized controlled trials, to evaluate the impact of new therapies.

SCAN-LEAF (Long-term Epidemiological Follow-up of Non-small Cell Lung Cancer in Scandinavia) is a retrospective, longitudinal study intended to describe the epidemiology, clinical care, and outcomes of patients with NSCLC in Scandinavia. The SCAN-LEAF project is currently based on the entire NSCLC population across Denmark and Sweden using data from national health care registries (Cohort 1) and from two select clinics in Sweden (Cohort 2). SCAN-LEAF is part of I-O Optimise, a multinational collaboration aimed at developing a research framework to provide timely insights into the evolving real-world management of thoracic malignancies.^22^

In this analysis of the SCAN-LEAF study, we provide insight into the preimmunotherapy baseline in Denmark and Sweden by reporting trends in overall survival (OS) in patients diagnosed with incident NSCLC between 2005 and 2015, using national registries data (Cohort 1).

## Materials and Methods

### Study Design and Database Overview

SCAN-LEAF is a retrospective cohort study. Swedish and Danish cohorts were established by linking national registries (The National Patient Register,^23^ the National Prescribed Drug Register [Sweden only],^24^ and the Cause of Death Register^25^^,^^26^) and included data on all inpatient and outpatient diagnoses of NSCLC. Data were retrieved on all adult patients diagnosed with incident NSCLC from January 1, 2005, to December 31, 2015, with follow-up from the date of first NSCLC diagnosis until death, emigration, or the end of the study period (December 31, 2016).

The National Cancer Registries in Denmark and Sweden have nationwide coverage and are updated annually. Reporting of newly diagnosed cancer cases into the Danish Cancer Registry has been mandatory since 1987. The Swedish Cancer Registry receives data from all oncology clinics across Sweden, and reporting is compulsory for every cancer diagnosed at clinical, morphologic, or other laboratory examinations and for cases diagnosed at autopsy.

### Study Population

The study was conducted in accordance with the International Society for Pharmacoepidemiology Guidelines for Good Epidemiology Practices and with the ethical principles set forth in the Declaration of Helsinki. The study protocol was approved by the Independent Ethics Committee of the lead institution (Karolinska) as required by local law before study initiation. This was a retrospective observational study using pseudonymized patient data from national registries. Patients were not contacted or directly affected by study participation, thus obtaining informed consent was not applicable.

Patients diagnosed with NSCLC (identified by an International Statistical Classification of Diseases and Related Health Problems, 10th revision [International Classification of Diseases or ICD-10] code for malignant neoplasm of bronchus and lung [C34] and an International Classification of Diseases for Oncology, third edition [ICD-O-3] code for NSCLC histology [Supplementary Table 1]) during the study inclusion period and aged 18 years or older at first diagnosis were included in this analysis. Patients with missing age/sex data or a concomitant primary tumor at diagnosis (i.e., within 5 y before NSCLC diagnosis), except for nonmetastatic nonmelanoma skin cancer (ICD-10 codes C44 and C4A), were excluded.

### Data Collection

In the Danish Cancer Registry, stage at diagnosis was based on the sixth edition of the TNM classification system^5^ before 2008 and the seventh edition^8^ starting in 2009. In Sweden, tumors were classified using the sixth edition of the TNM classification system before 2010 and the seventh edition thereafter. Mortality data (date of death) for OS were retrieved from the respective Cause of Death Registers in Denmark and Sweden according to ICD-10 codes.

### Data Analysis and Statistical Methodology

Patient characteristics are presented using summary statistics for the overall study period (2005–2015) and for individual years. Temporal trends in TNM staging and tumor histology were evaluated descriptively on the basis of annual percentages. For data on patient age and comorbidities, median and interquartile ranges were calculated. For all other patient characteristics, total numbers and percentages were calculated.

OS was defined as the time from initial NSCLC diagnosis to death from any cause during the observation period. Kaplan-Meier methodology was used to estimate OS probability (95% confidence interval [CI]) at 1, 3, and 5 y by histological subtype (NSQ or squamous cell carcinoma [SQ]), TNM stage, and year of diagnosis. Changes in OS were evaluated using the Cochrane-Armitage test for trends in the proportion of survivors in time, performed at 1, 3, and 5 y postdiagnosis. Statistical significance was evaluated using two-sided tests with an alpha level of 0.05. Prognostic factors associated with risk of death within 2 years after diagnosis in patients with stage IIIB or IV NSCLC were evaluated using a Cox proportional hazards regression model. Fully adjusted models by country are presented with hazard ratio (95% CI) and corresponding *p* values for all model covariates. The variables included in the models (disease stage, age, sex, histology, and year of diagnosis) were selected a priori on the basis of clinician recommendation. Statistical analyses were performed using SAS version 9.3 (SAS Institute, Cary, NC).

## Results

### Database Overview

Overall, 30,067 patients Sweden and 31,939 patients from Denmark who were diagnosed with incident NSCLC between 2005 and 2015 were included (Supplementary Fig. 1).

### Patients

Demographic and clinical characteristics of patients diagnosed with NSCLC in the study period are shown in Table 1 and Supplementary Tables 2 to 5. Between 2005 and 2015, the number of patients diagnosed annually increased by 18% (2482–2934) in Sweden and by 26% (2496–3135) in Denmark. For patients from Sweden and Denmark, median age at diagnosis in the study period was 70.0 and 69.0 y, approximately half were male (51.0% and 52.0%), and 18.9% and 14.0% had chronic pulmonary disease, respectively. Over time, the median age at diagnosis increased (Sweden: 69.0–71.0 y [Supplementary Table 2]; Denmark: 68.0–70.0 y [Supplementary Table 3]), whereas the proportion of males decreased over the same period (Sweden: 55.2%–48.6% [Supplementary Table 2]; Denmark: 53.9%–50.6% [Supplementary Table 3]).

**Table 1:** Demographic and Clinical Characteristics of the Incident NSCLC Population in Sweden and Denmark

Characteristic	Sweden			Denmark		
	All	NSQ	SQ	All	NSQ	SQ
	(N = 30,067)	(N = 18,157)	(N = 7134)	(N = 31,939)	(N = 17,386)	(N = 8457)
Age at NSCLC diagnosis, y Median (Q1–Q3)	70 (63–76)	69 (63–76)	72 (65–78)	69 (62–76)	67 (61–75)	71 (65–77)
Sex						
Male, n (%)	15,320 (51.0)	8312 (45.8)	4444 (62.3)	16,593 (52.0)	7794 (44.8)	5510 (65.2)
Comorbidities, n (%),^a^						
Chronic pulmonary disease	5670 (18.9)	2953 (16.3)	1789 (25.1)	4475 (14.0)	2080 (12.0)	1460 (17.3)
Congestive heart failure	2537 (8.4)	1389 (7.6)	724 (10.1)	1128 (3.5)	543 (3.1)	364 (4.3)
TNM classification at diagnosis, n (%)						
IA	2771 (9.2)	2037 (11.2)	548 (7.7)	2003 (6.3)	1279 (7.4)	516 (6.1)
IB	2002 (6.7)	1133 (6.2)	684 (9.6)	2135 (6.7)	1212 (7.0)	690 (8.2)
IIA	679 (2.3)	390 (2.1)	222 (3.1)	841 (2.6)	429 (2.5)	321 (3.8)
IIB	1080 (3.6)	491 (2.7)	442 (6.2)	1481 (4.6)	688 (4.0)	614 (7.3)
IIIA	2709 (9.0)	1335 (7.4)	926 (13.0)	3594 (11.3)	1672 (9.6)	1407 (16.6)
IIIB	3996 (13.3)	2008 (11.1)	1263 (17.7)	3735 (11.7)	1625 (9.3)	1371 (16.2)
IV	14,544 (48.4)	9453 (52.1)	2489 (34.9)	16,486 (51.6)	9630 (55.4)	3068 (36.3)
Missing	2286 (7.6)	1310 (7.2)	560 (7.8)	1664 (5.2)	851 (4.9)	470 (5.6)
Histology, n (%)						
Nonsquamous NSCLC	18,157 (60.4)	18,157 (100)	0	17,386 (54.4)	17,386 (100)	0
Adenocarcinoma	17,097 (56.9)	17,097 (94.2)	0	16,917 (53.0)	16,917 (97.3)	0
Large cell carcinoma	1060 (3.5)	1060 (5.8)	0	469 (1.5)	469 (2.7)	0
Squamous cell carcinoma	7134 (23.7)	0	7134 (100)	8457 (26.5)	0	8457 (100)
NSCLC NOS	4119 (13.7)	0	0	4675 (14.6)	0	0
Other miscellaneous NSCLC	657 (2.2)	0	0	1421 (4.4)	0	0

NOS, not otherwise specified; NSQ, nonsquamous cell carcinoma; Q, quartile; SQ, squamous cell carcinoma.

a In the Swedish registry, the full lookback period was available. In the Danish registry, the lookback period was 2 years.

The most common histological subtypes were NSQ (60.4%; including adenocarcinoma, 56.9%) and SQ (23.7%) in Sweden, with similar trends observed in Denmark (NSQ: 54.4%; adenocarcinoma 53.0%; SQ 26.5%). Over time, the proportion of patients diagnosed with NSQ histology gradually increased (Sweden: 51.8%–66.7%; Denmark: 45.2%–60.5%), whereas those diagnosed with not otherwise-specified histology decreased (Sweden: 19.6%–8.4%; Denmark: 25.5%–8.6% [Supplementary Tables 2 and 3]). Overall, patients with SQ histology were slightly older than those with NSQ histology (Sweden: 72 versus 69 y; Denmark: 71 versus 67 y), and a higher proportion was male (Sweden: 62.3%; Denmark: 65.2%) than patients with NSQ histology (45.8% and 44.8%, respectively).

**Figure 1:**
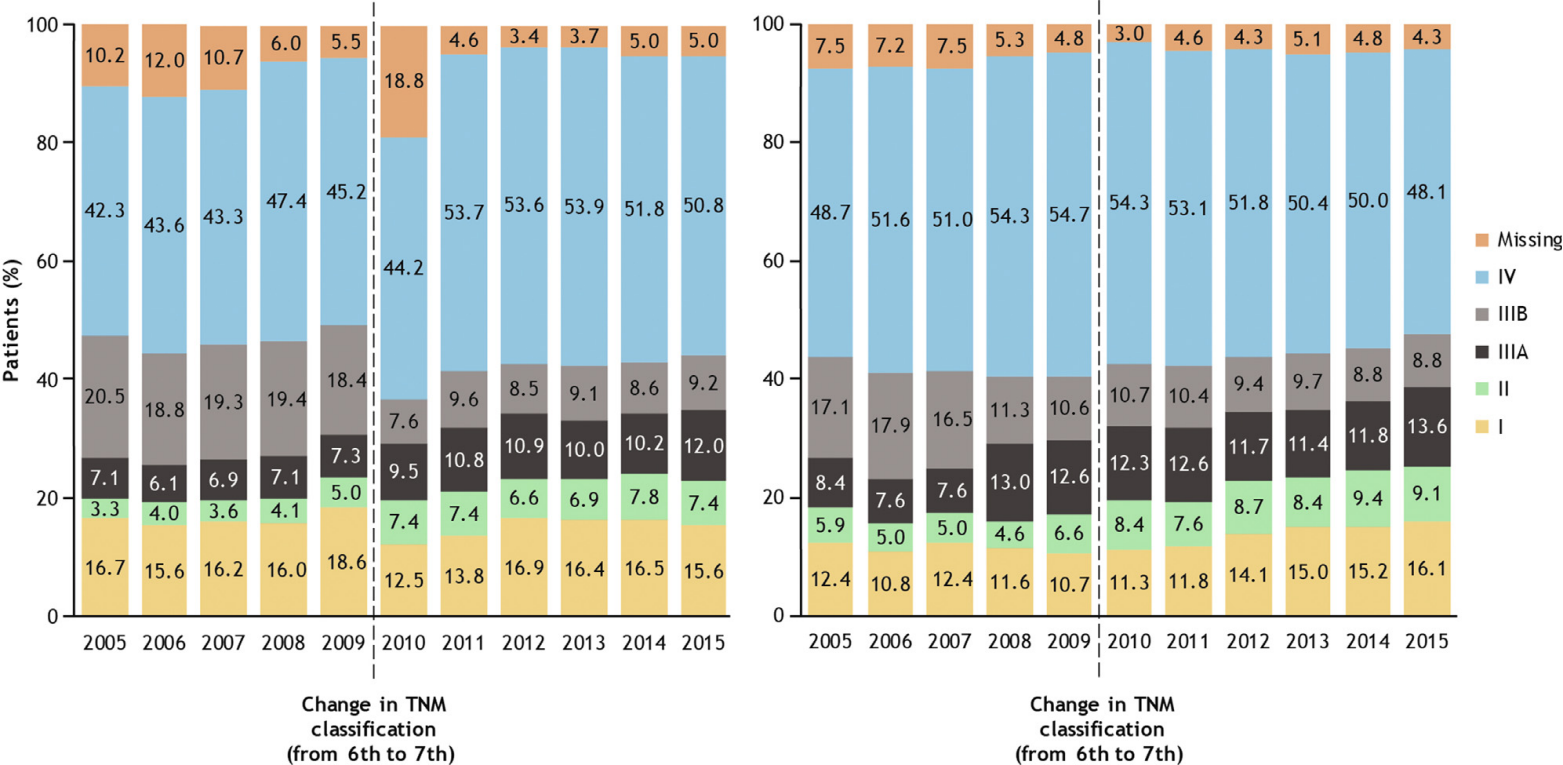
Evolution of TNM stage distribution at diagnosis between 2005 and 2015 in (*A*) Sweden and (*B*) Denmark.

Approximately half of the patients with NSCLC from Sweden (48.4%) and Denmark (51.6%) had metastatic stage IV disease at diagnosis (Table 1). Most patients with NSQ histology (Sweden: 52.1%; Denmark: 55.4%) and approximately one-third of those with SQ histology (Sweden: 34.9%; Denmark: 36.3%) were diagnosed with stage IV NSCLC. Over the analysis period, the proportion of patients diagnosed with stage IV NSCLC remained high in both countries (Fig. 1, Supplementary Tables 2 and 3). Approximately one-fifth of the patients with NSQ histology and one-third of those with SQ histology were diagnosed at stage III (Table 1). The proportion of patients diagnosed with stage IIIA NSCLC gradually increased over time in both countries (Sweden: 7.1%–12.0%; Denmark: 8.4%–13.6%), whereas the proportion diagnosed with stage IIIB generally declined (Sweden: 20.5%–9.2%; Denmark: 17.1%–8.8%; Fig. 1). Diagnoses of stage II NSCLC increased over time in both Sweden (3.2%–7.4%) and Denmark (5.9%–9.1%), whereas there was no notable change in the diagnoses of stage I NSCLC in either country in the study period.

### Evolution of OS, Overall, and by Histology

Over the study period, there were significant improvements in OS in both Sweden and Denmark (Supplementary Fig. 2). In Sweden, 1-year OS (95% CI) increased from 38% (36–40) in 2005 to 49% (47–51) in 2015; 3-year OS increased from 17% (15–18) in 2005 to 23% (21–25) in 2013; and 5-year OS increased from 12% (11–13) in 2005 to 16% (14–17) in 2011 (all *p* < 0.0001).

Similarly, in Denmark, 1-year OS (95% CI) increased from 37% (35–39) in 2005 to 49% (47–51) in 2015; 3-year OS increased from 16% (14–17) in 2005 to 24% (23–26) in 2013; and 5-year OS increased from 11% (10–12) in 2005 to 15% (14–17) in 2011 (all *p* < 0.0001).

Significant improvements in OS were observed in both countries regardless of histological subtype (NSQ and SQ; Supplementary Fig. 1).

### OS by Disease Stage

**Figure 2:**
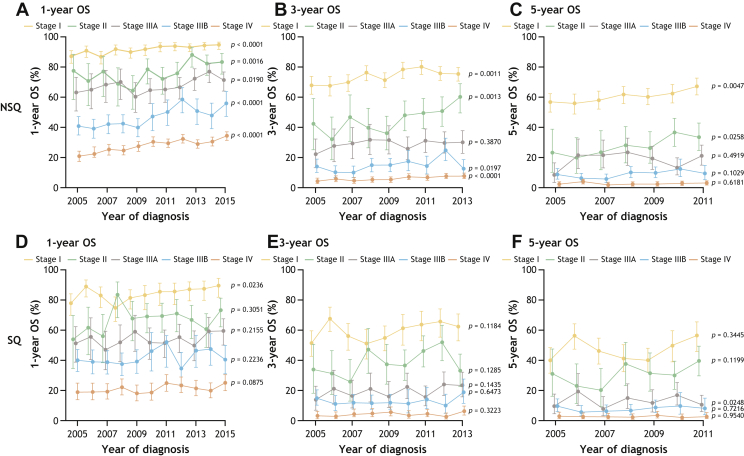
OS at 1 year (*A* and *D*), 3 years (*B* and *E*), and 5 years (*C* and *F*) in patients with incident NSCLC in Sweden diagnosed from 2005 to 2015 by stage and histology (NSQ: *A–C*; SQ: *D–F*). *p* value less than 0.05 indicates a significant trend over time. NSQ, nonsquamous; OS, overall survival; SQ, squamous.

**Figure 3:**
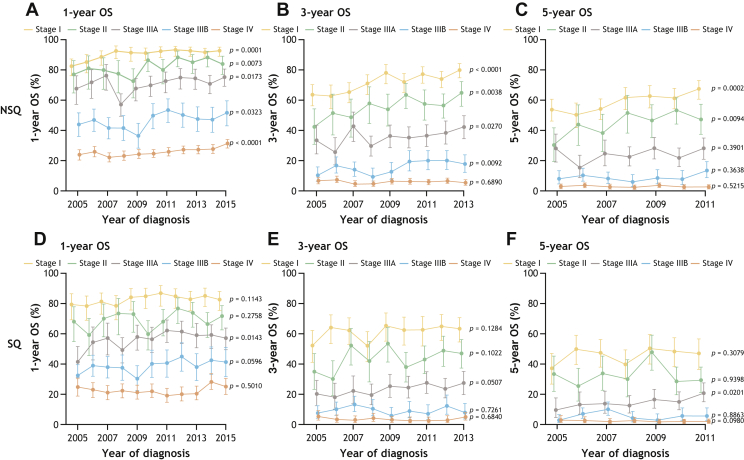
OS at 1 year (*A* and *D*), 3 years (*B* and *E*), and 5 years (*C* and *F*) in patients with incident NSCLC in Denmark diagnosed from 2005 to 2015 by stage and histology (NSQ: *A–C*; SQ: *D–F*). *p* value less than 0.05 indicates a significant trend over time. NSQ, nonsquamous; OS, overall survival; SQ, squamous.

In both countries, OS at 1 year, 3 years, and 5 years declined with increasing TNM stage at diagnosis, regardless of histology (Figs. 2 and 3).

**Stage I NSCLC.** Among patients with stage I NSQ histology, 1-year OS (95% CI) increased significantly from 2005 to 2015 (Figs. 2*A* and 3*A*; Sweden: 87% [83–91] to 95% [92–97]; *p* < 0.001; Denmark: 82% [76–88] to 92% [89–95]; *p* < 0.001). Marked survival improvements were observed in the period from 2005 to 2011 in Sweden and from 2005 to 2008 in Denmark. Three- and 5-year OS significantly improved over the analysis period (Figs. 2*B* and *C* and 3*B* and *C*), with 5-year OS (95% CI) increasing from 56% (50–62) to 67% (62–73; *p* = 0.004) in Sweden and from 53% (46–61) to 67% (61–73; *p* < 0.001) in Denmark.

In patients with stage I SQ histology, significant improvements in 1-year OS (95% CI) were observed in Sweden (Fig. 2*D*; 77% [70–85] to 89% [83–95]; *p* = 0.024), whereas only modest improvements occurred in Denmark (Fig. 3*D*). Changes in 3- and 5-year OS over time were not statistically significant in patients with stage I SQ histology (Figs. 2*E* and *F* and 3*E* and *F*). The 5-year OS (95% CI) for patients diagnosed in 2011 (the latest year available) was 55% (46–67) in Sweden and 47% (38–58) in Denmark.

**Stage II NSCLC.** Among patients with stage II NSQ histology, 1-year OS (95% CI) significantly increased between 2005 and 2015 (Fig. 2*A*; Sweden: 77% [64–94] to 83% [77–90]; *p* = 0.002; Fig. 3*A*; Denmark: 77% [67–88] to 83% [78–90]; *p* = 0.007). Significant improvements in 3- and 5-year OS over time were also observed (Figs. 2*B* and *C* and 3*B* and *C*). Notably, 5-year OS (95% CI) increased from 23% (12–43) to 33% (25–44; *p* = 0.03) in Sweden and from 30% (20–43) to 47% (38–58; *p* = 0.01) in Denmark over the study period.

In patients with stage II SQ histology, no significant trends in 1-, 3-, or 5-year OS were observed at any time point in either country (Figs. 2*D*–*F* and 3*D–F*).

**Stage IIIA NSCLC.** In patients with stage IIIA NSQ histology, 1-year OS (95% CI) improved over the study period (Fig. 2*A*; Sweden: 63% [53–75] to 71% [65–78]; *p* = 0.02; Fig. 3*A*; Denmark: 67% [58–77] to 75% [69–81]; *p* = 0.02). An increase in 3-year OS (95% CI) was observed for patients from Denmark only (Fig. 3*B*; 33% [25–44] to 42% [35–50]; *p* = 0.03).

In patients with stage IIIA SQ histology from Denmark (Fig. 3*D–F*), increases in OS (95% CI) in the study period reached significance at 1 year (42% [33–54] to 57% [51–65]; *p* = 0.01) and 5 years (10% [5–19] to 21% [15–28]; *p* = 0.02). Overall, no notable trends in OS were observed over time in patients with stage IIIA SQ between 2005 and 2011; however, there were some temporal improvements in both countries (Figs. 2*D–F* and 3*D–F*).

**Stage IIIB NSCLC.** Among patients with stage IIIB NSQ histology, there was a significant improvement in 1-year OS (95% CI) over time (Fig. 2*A*; Sweden: 41% [35–48] to 56% [48–65]; *p* < 0.001; Fig. 3*A*; Denmark: 43% [36–52] to 51% [43–60]; *p* = 0.03), with the greatest increases in OS observed between 2009 and 2011. Improvements in 3-year OS (95% CI) were observed for patients in Denmark (Fig. 3*B*; 10% [6–16] to 17% [12–25]; *p* = 0.01). In Sweden, although there were some temporal improvements in OS over the study period, the OS was similar in 2005 and 2011 (Fig. 2*B*).

No significant changes in OS were observed for patients with stage IIIB SQ histology (Figs. 2*D–F* and 3*D–F*).

**Stage IV NSCLC.** In patients with stage IV NSQ histology, 1-year OS (95% CI) significantly improved over time in both Sweden (Fig. 2*A*; 21% [18–24] to 34% [31–37]; *p* < 0.001) and Denmark (Fig. 3*A*; 23% [20–27] to 31% [28–34]; *p* < 0.001). Overall, there were no significant trends in 3- and 5-year OS over the analysis period in this patient subgroup, although some temporal changes occurred in patients with NSQ histology from Sweden (*p* < 0.001; Figs. 2*B* and *C* and 3*B* and *C*).

Among patients with stage IV SQ histology, no changes in 1-, 3-, and 5-year OS were observed over time (Figs. 2*D–F* and 3*D–F*).

### OS According to Age

Regardless of disease stage, tumor histology, or country, median OS and 1-year OS declined with increasing patient age (Table 2*A* and *B*).

**Table 2:** OS (mo) in (*A*) Swedish and (*B*) Danish Patients Diagnosed With NSCLC by Age

	Incident Stage I–IIIA NSCLC						Incident Stage IIIB–IV NSCLC					
	NSQ			SQ			NSQ			SQ		
Age, y	N	Median OS (Q1–Q3), mo	1-y OS Probability (95% CI)	N	Median OS (Q1–Q3), mo	1-y OS Probability (95% CI)	N	Median OS (Q1–Q3), mo	1-y OS Probability (95% CI)	N	Median OS (Q1–Q3), mo	1-y OS Probability (95% CI)
<65	1632	83.5 (25.8–NE)	0.89 (0.88–0.91)	621	42.8 (16.0–NE)	0.81 (0.78–0.85)	3799	8.3 (3.5–18.1)	0.37 (0.35–0.38)	931	7.9 (3.6–16.8)	0.36 (0.33–0.39)
65–74	2269	57.0 (20.5–NE)	0.85 (0.84–0.87)	1143	34.7 (11.5–105.4)	0.74 (0.71–0.76)	4377	6.9 (2.7–15.4)	0.32 (0.31–0.34)	1464	6.2 (2.5–13.5)	0.28 (0.26–0.30)
≥75	1485	31.1 (12.1–76.9)	0.75 (0.73–0.77)	1058	16.4 (7.0– 46.3)	0.59 (0.56–0.62)	3285	4.9 (2.0–11.9)	0.25 (0.23–0.26)	1357	5.0 (2.2–10.4)	0.21 (0.19–0.23)

	Incident Stage I–IIIA NSCLC						Incident Stage IIIB–IV NSCLC					
	NSQ			SQ			NSQ			SQ		
Age, y	N	Median OS (Q1–Q3), mo	1-y OS Probability (95% CI)	N	Median OS (Q1–Q3), mo	1-y OS Probability (95% CI)	N	Median OS (Q1–Q3), mo	1-y OS Probability (95% CI)	N	Median OS (Q1–Q3), mo	1-y OS Probability (95% CI)
<65	1982	76.3 (23.6–NE)	0.88 (0.86–0.89)	796	37.7 (13.7–NE)	0.77 (0.74–0.80)	4462	7.4 (3.0–16.3)	0.33 (0.32–0.35)	1135	8.1 (3.4–16.0)	0.33 (0.31–0.36)
65–74	2028	55.0 (18.6–118.7)	0.83 (0.82–0.85)	1464	32.6 (11.4–99.6)	0.74 (0.71–0.76)	4035	5.9 (2.3–13.6)	0.28 (0.26–0.29)	1726	6.8 (2.9–14.0)	0.29 (0.27–0.32)
≥75	1270	27.7 (10.9–76.9)	0.72 (0.70–0.75)	1288	17.1 (6.9–49.1)	0.59 (0.57–0.62)	2758	4.2 (1.7–10.4)	0.21 (0.20–0.23)	1578	5.0 (2.1–10.5)	0.21 (0.19–0.23)

CI, confidence interval; NE, not estimable; NSQ, nonsquamous; OS, overall survival; Q, quartile; SQ, squamous.

### Prognostic Factors for Risk of Death

**Figure 4:**
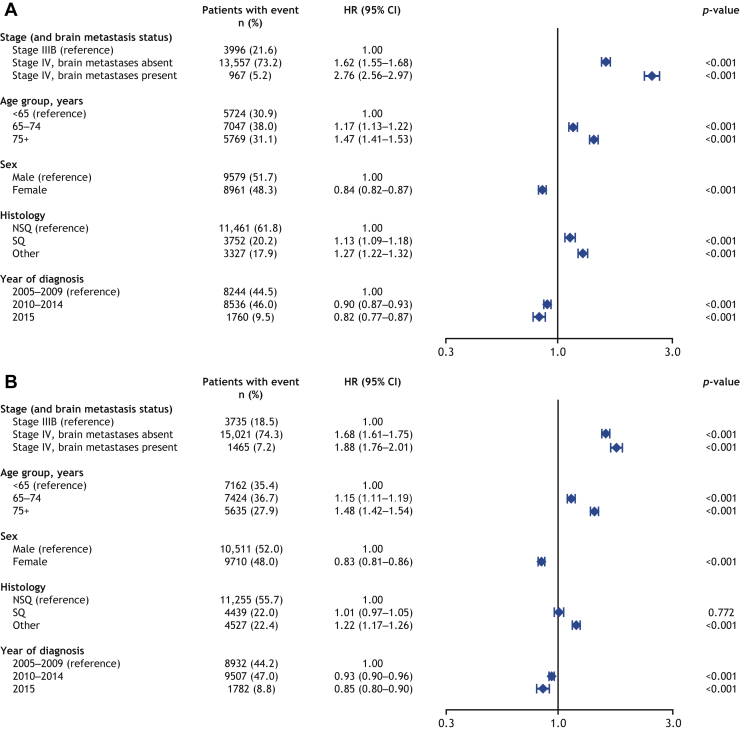
Prognostic factors for OS over a 2-year follow-up period in (*A*) Swedish and (*B*) Danish patients diagnosed with locally advanced or metastatic NSCLC at diagnosis (stage IIIB–IV). CI, confidence interval; HR, hazard ratio; NSQ, nonsquamous; OS, overall survival; SQ, squamous.

Cox modeling analysis of factors associated with OS in the 2 years after diagnosis of stage IIIB or IV NSCLC in Sweden (n = 18,540) and Denmark (n = 20,221) revealed that younger age, female sex, NSCLC stage IIIB versus IV, and more recent diagnosis were significantly associated with improved OS (*p* < 0.001) (Fig. 4*A* and *B*). In Sweden, but not Denmark, NSQ histology was significantly associated with longer survival compared with SQ histology (*p* < 0.001).

## Discussion

To evaluate the potential real-world benefits of newer treatments for lung cancer, an understanding of the disease landscape before their availability is essential. As part of the wider I-O Optimise initiative, SCAN-LEAF constitutes the largest known real-world study of patients with lung cancer in Scandinavia using national-level data and will provide a comprehensive baseline picture of outcomes for patients with NSCLC in this region. In this analysis, we report the characteristics and OS trends for patients diagnosed with incident NSCLC before the reimbursement and widespread use of immunotherapies.

The rate of new NSCLC diagnoses between 2005 and 2015 increased by 18% in Sweden and 26% in Denmark. The demographic and clinical characteristics of patients at diagnosis were generally consistent with data from previous real-world studies in Europe.^27^, ^28^, ^29^, ^30^ The decreased proportion of patients diagnosed with NSCLC NOS between 2005 and 2015 was consistent with other reports and likely reflects improvement in the accuracy of histology specification in the past decade. Such improvements in histology specification have been associated with consequent changes in the use of systemic anticancer therapies.^31^

Accurate staging of NSCLC, using an evolving TNM staging system, is key to determining optimal management pathways for patients. Here, some temporal changes were observed in the distribution of TNM stage, and hence disease severity, at diagnosis. These changes were partly related to the introduction of the seventh edition of the TNM classification system^6^ during the study period, which was adopted in 2009 in Denmark and in 2010 in Sweden. The seventh edition provided a more accurate correlation between TNM stage and survival statistics than the sixth edition, by revising tumor size cutoffs for the T descriptor and acknowledging the importance of pleural effusions and mediastinal invasion for the M descriptor^.^^5^^,^^6^^,^^32^ These revisions, along with changes to the stage groups, led to the upstaging of some stage IB tumors to stage IIA and IIB and stage IIIB with pleural effusion to stage IV, and the downstaging of some IIIB tumors to stage IIB and IIIA (multiple tumor nodules in same lobe changed from T4 to T3) and some stage IV tumors to stage IIIA/B (multiple tumor nodules in different lobes but the same lung were changed from M1 to T4).^6^

This may partly explain the increased proportion of patients diagnosed with stage IV in Sweden, and with IIIA NSCLC overall, and the decreased proportion diagnosed with stage IIIB around the time the seventh edition was adopted. Changes introduced in the eighth edition in 2018 are likely to enhance prognostic stratification, which could affect the future distribution of NSCLC stages.^33^ In this analysis, after the adoption of the TNM seventh edition, a slight decline was observed in the proportion of patients diagnosed with stage IV NSCLC. This was most pronounced in Denmark and may reflect the positive impact of the Cancer Patient Pathways, implemented nationally in 2009^34^ to fast-track diagnosis and provide more coordinated treatment plans for patients with suspected malignancy.

In 2015, the majority of incident NSCLC diagnoses remained at the metastatic stage. Several recent real-world studies in European countries have revealed similar rates of locally advanced or metastatic disease at diagnosis,^28^^,^^29^^,^^35^ emphasizing the need for the detection of NSCLC at earlier stages. On the basis of the results of the US National Lung Screening Trial^36^ and several pilot studies in Europe, the European Union Lung Cancer Screening Implementation Group set forth recommendations for the implementation of low-dose computed tomography (CT) screening for the detection of early lung cancer in a 2017 position statement.^37^^,^^38^ Indeed, implementation of lung cancer CT screening through pilot studies has been suggested in both Sweden and Denmark.^39^ Furthermore, 10-year results from the large Dutch/Belgian NELSON trial reported that CT screening reduced the risk for mortality owing to lung cancer by 26% in high-risk male individuals and 33% in a small subgroup of females compared with those in a no-screening control group.^40^ Nevertheless, despite the apparent benefits, concerns on radiation exposure and false-positive results are important considerations for the implementation of widespread screening.^39^^,^^41^

Regarding the evolution of OS in NSCLC (all stages) between 2005 and 2015, our study revealed a significant improvement overall and in patients with NSQ and SQ histology separately. A study based on the Surveillance, Epidemiology, and End Results program in the United States also found a decline in mortality owing to NSCLC between 2006 to 2016, which corresponded to the timing of approvals for targeted therapies.^42^ Thus, as TKIs were first launched for the treatment of NSCLC in Sweden and Denmark in 2010, it is likely that the approval of targeted therapies over the period of our study also affected the OS patterns observed.

Furthermore, our study revealed that changes in survival over the study period varied according to the disease stage at diagnosis and tumor histology, with less improvement in OS observed in patients with SQ versus NSQ histology, for all stages. Among patients with stage I and II NSCLC, significant improvement in 5-year OS was observed in both countries among those with NSQ disease only. This may be related to the increased use of stereotactic body radiation therapy (SBRT) in medically inoperable patients during this period (Sørensen et al. personal communication) as well as improvements in surgical technology and techniques.^41^ The difference in OS according to histology may also reflect the inconsistent impact of SBRT according to histologic subtype; SQ disease has been associated with lower survival after SBRT in early stage NSCLC.^43^^,^^44^ In addition, although data on smoking were not collected, changing OS rates over time are likely to reflect changes in smoking patterns in Sweden and Denmark, particularly between sexes, over the preceding decades.^45^^,^^46^

In our study, 1-year OS seemed to improve for patients with stage IIIA NSQ histology after adoption of the seventh TNM classification. Some improvement in 3-year OS for patients with stage IIIA disease was observed in Denmark, possibly reflecting the impact of the Cancer Patient Pathway since 2009; this may increase survival through the earlier initiation of treatment and more appropriate follow-up.^34^ Overall, there were some improvements in 3- and 5-year OS for patients with stage IIIA SQ disease in Denmark and 1-year OS for patients with stage IIIB NSQ and SQ disease, particularly from 2010 onward.

Treatment of stage III NSCLC remains difficult and controversial, mainly owing to heterogeneity regarding tumor size and location and lymph node involvement. Improvements in the therapeutic management of patients with stage IIIA disease and the ability to treat eligible patients with chemoradiation may have contributed to the OS patterns (Sørensen et al., in preparation). However, the effectiveness of treatments used in the stage IIIB population remained suboptimal because less than 20% of patients diagnosed in 2013 were alive 3 years after diagnosis. Similar improvements in 1-year OS results for patients aged 65 years and older diagnosed with stage IIIB NSCLC during 2009 to 2013 have been reported from the Netherlands Cancer Registry.^47^

Improvements in OS for patients with stage IV NSCLC were limited to 1- and 3-year OS and were only observed in patients with NSQ disease. This might reflect changes in therapeutic options, with the availability of targeted therapy for patients with *EGFR/ALK* aberrations and some improvement in the proportion of patients with stage IV receiving SACT after diagnosis in the past decade (Sørensen et al. personal communication). Data from the Netherlands Cancer Registry reported survival benefits for patients with stage IV NSCLC within the first year of diagnosis from 2004 to 2009 compared with 1989 to 1993; however, this effect disappeared after adjusting for changes in treatment in time, indicating this may have driven the observed improvement.^48^ In addition, in the Netherlands, increased use of chemotherapy was associated with improved OS among patients with stage IV NSCLC between 2001 and 2012. Patients with SQ disease were less likely to receive chemotherapy compared with those with NSQ disease.^49^

Over the study period, it is likely that improvements in imaging led to improved staging, as CT scans and positron emission tomography–CT scans became more widely available. Therefore, the changes in OS observed by stage in the study period may be attributed to both changes in therapeutic management and the evolution of staging during that time (so-called Will-Rogers phenomenon).^50^ While the standard of care evolved and treatment recommendations for nonmetastatic patients changed between 2005 and 2015 with the introduction of SBRT and chemoradiation as curative option in nonresectable patients, no major changes occurred regarding therapeutic options for advanced patients, except those with *EGFR/ALK* aberrations. Reflecting the lack of significant improvement in managing NSCLC, the 3-year survival rate of metastatic patients was still approximately 5% in both countries. The availability of new treatment options, such as immunotherapies and newer TKIs, is expected to have improved survival outcomes for patients with advanced NSCLC post-2015. Future analyses of the SCAN-LEAF database will evaluate treatment patterns and outcomes over time in Denmark and Sweden.

The prognostic factors identified in this analysis, including younger age, NSQ histology, and earlier stage, are similar to those identified in a national patient registry study of lung cancer in Germany.^45^ In that study, across all age groups, sex was an independent predictor of death after adjustment for histology and staging (males had a greater risk of death than females). The observation of worse survival outcomes with increasing patient age is also consistent with previous reports^40^^,^^46^ and may reflect the undertreatment of older patients.^40^

SCAN-LEAF uses nationwide, high-quality cancer and death registries from Sweden and Denmark, including a large, unselected population of patients diagnosed with NSCLC. These registries have been available for approximately 20 years, allowing the evolution of NSCLC diagnosis, TNM staging, and outcomes to be analyzed in time. Thus, the data used here make these results highly generalizable to the population of Scandinavia. As physicians had the option to back-date the diagnosis date, for example, based on the first suspected date of lung cancer, this may have reduced the diagnostic accuracy leading to possible misdiagnoses. In addition, an important limitation of our study is the change in the TNM staging system during the analysis period, which makes OS comparisons between patients diagnosed before and after the change challenging. A further limitation of this study is that data on the SACT treatment received and clinical information, such as Eastern Cooperative Oncology Group performance status, smoking history, and biomarker status, were not available in the registries, limiting the scope of the analyses at a national level. In addition, data on brain metastases were inconsistently captured by the registries. Finally, in the Swedish registry, the full lookback period was available, including index date, to identify comorbidities, whereas in the Danish registry, the lookback period was available for 2 years. Therefore, the proportion of patients with comorbid conditions seemed disproportionality higher in the cohort from Sweden versus Denmark.

This analysis of the SCAN-LEAF study provides valuable insights into the characteristics of patients diagnosed with NSCLC between 2005 and 2015 in Sweden and Denmark, and survival trends in a period when staging systems, diagnostic techniques, and early diagnosis strategies for these patients were evolving. Despite survival improvements in the study period for some groups of patients, an unmet need remains especially for patients with stage IV NSCLC and for those with SQ histology. Future analyses from the SCAN-LEAF project will evaluate the potential impact of increased use of new TKIs and ICIs on OS.
