# Supplementary material for: Epidemiology and Survival Outcomes for Patients With NSCLC in Scandinavia in the Preimmunotherapy Era: A SCAN-LEAF Retrospective Analysis From the I-O Optimise Initiative
Source: JTO Clin Res Rep. 2021 Mar 24;2(5):100165. doi: 10.1016/j.jtocrr.2021.100165 (PMC8474201; doi:10.1016/j.jtocrr.2021.100165)
Supplement: Supplementary Material [file mmc1.docx]

**SUPPLEMENTAL DIGITAL CONTENT**

**Epidemiology and Survival Outcomes for Patients With NSCLC in Scandinavia in the Pre-Immunotherapy Era: A SCAN-LEAF Retrospective Analysis From the I-O Optimise Initiative**

Simon Ekman, MD, PhD, Assoc. Professor^a^ Pia Horvat, PhD^b^ Mats Rosenlund, PhD^c,d^ Anne Mette Kejs, MSc^e^ Dony Patel, PhD^b^ Ariadna Juarez-Garcia, PhD^f^ Laure Lacoin, PharmD^g^ Melinda J Daumont, PhD^h^ John R. Penrod,^i^ Odd Terje Brustugun, MD, PhD^j^ Jens Benn Sørensen, Assoc. Professor^k^

^a^Thoracic Oncology Center, Karolinska University Hospital/Department of Oncology-Pathology, Karolinska Institutet, Stockholm, Sweden

^b^Real-World Evidence Solutions, IQVIA, London, UK

^c^Real-World & Analytics Solutions, IQVIA, Solna, Sweden

^d^Department of Learning, Informatics, Management and Ethics (LIME), Karolinska Institutet, Stockholm, Sweden

^e^Real-World & Analytics Solutions, IQVIA, Copenhagen, Denmark

^f^Worldwide Health Economics & Outcomes Research, Bristol Myers Squibb, Uxbridge, UK

^g^EPI-FIT, Bordeaux, France

^h^Worldwide Health Economics & Outcomes Research, Bristol Myers Squibb, Braine-L’Alleud, Belgium

^i^Worldwide Health Economics & Outcomes Research, Bristol Myers Squibb, Princeton, New Jersey

^j^Section of Oncology, Drammen Hospital, Vestre Viken Hospital Trust, Drammen, Norway

^k^Department of Oncology, Rigshospitalet, Copenhagen, Denmark

**SUPPLEMENTARY APPENDIX**

**Supplementary Figure 1. Patient disposition. *Except for non-metastatic skin cancer or benign tumors (pre-specified).**


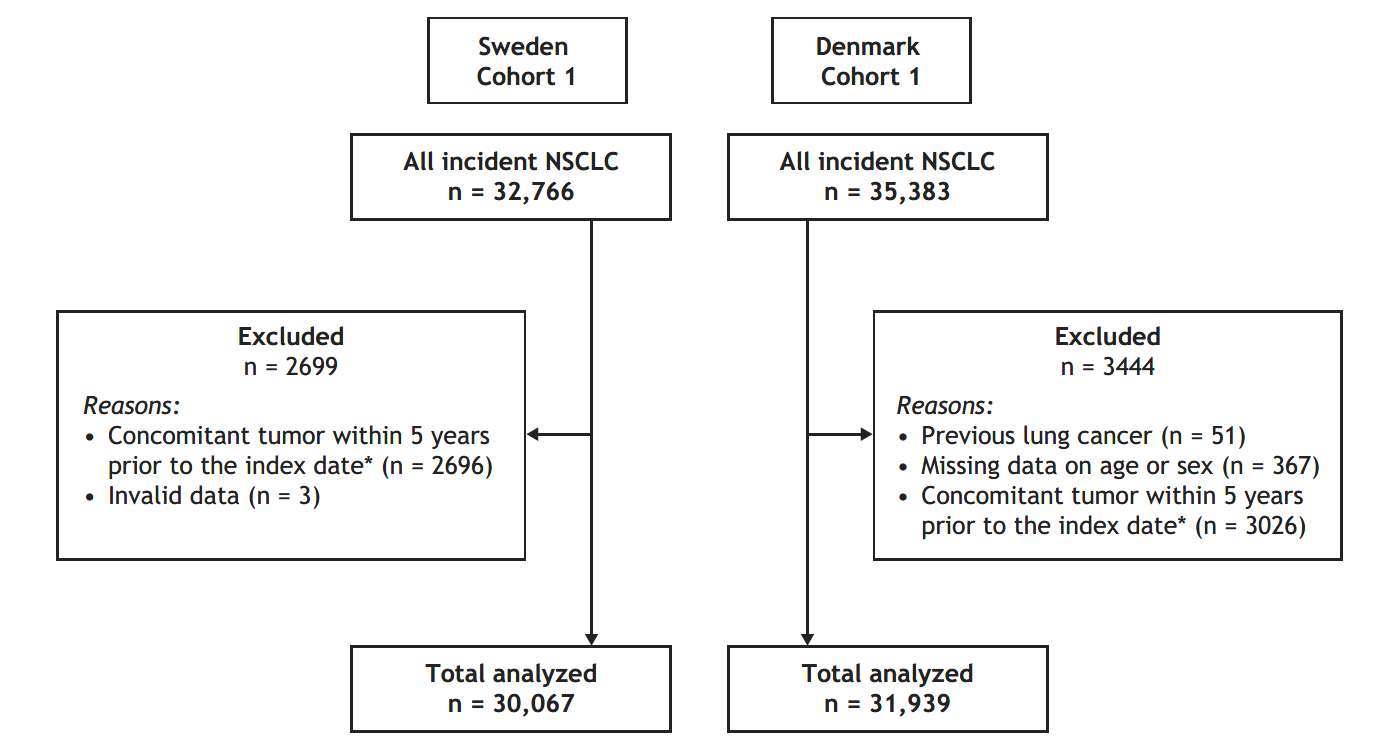


**Supplementary Figure 2. OS over time in patients with incident NSCLC in (A) Sweden and (B) Denmark diagnosed from 2005–2015 overall and by histology. *p* < 0.05 indicates a significant trend over time. NSCLC, non-small cell lung cancer; NSQ, non-squamous; OS, overall survival; SQ, squamous.**


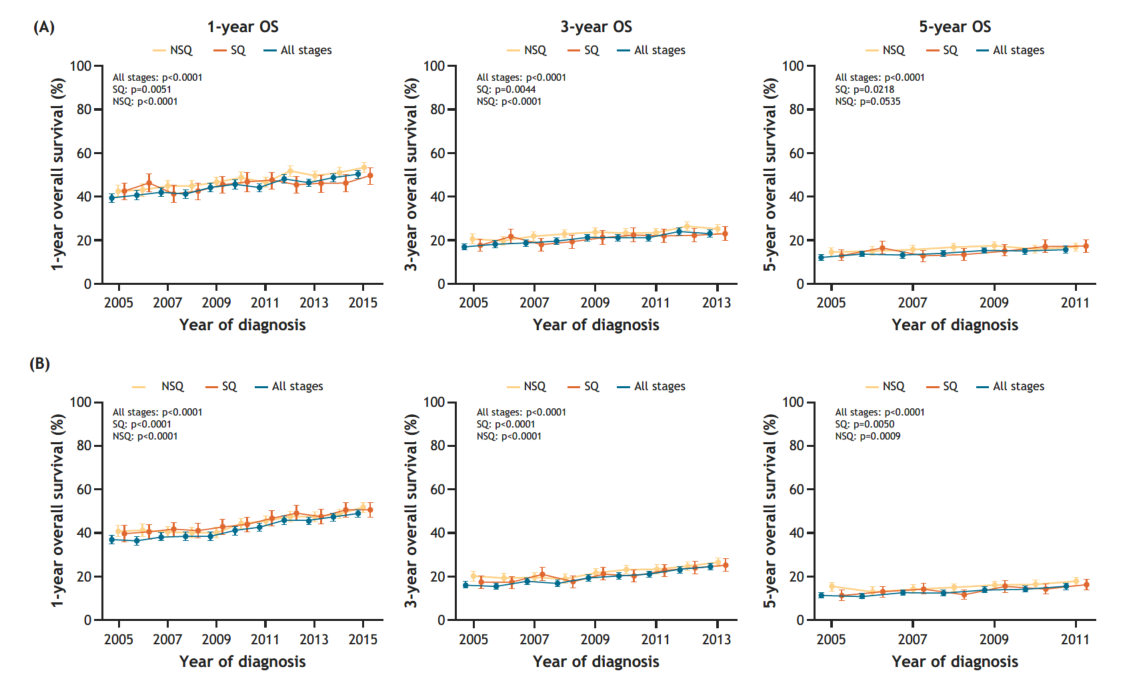


**Supplementary Table 1. ICD-10 Morphology Codes for NSCLC**

| **Morphology code (ICD-O/3)** | **Type of NSCLC** |
| --- | --- |
|  | **Adenocarcinoma (non-squamous NSCLC)** |
| 81403 | Adenocarcinoma UNS |
| 81443 | Enteric adenocarcinoma |
| 82303 | Solid adenocarcinoma with mucin production |
| 82443 | MANEC mixed adenoneuroendocrine carcinoma |
| 82500 | Atypical adenomatous hyperplasia |
| 82502 | Adenocarcinoma in situ, non-mucinous |
| 82503 | Adenocarcinoma, bronchio-alveolar carcinoma, bronchiolar carcinoma, (incl pathologic in situ-variant) |
| 82523 | Bronchio-alveolar carcinoma |
| 82532 | Adenocarcinoma in situ, mucinous |
| 82533 | Adenocarcinoma, mucinous bronchiolo-alveolar carcinoma |
| 82543 | Bronchio-alveolar carcinoma, mixed mucinous and non-mucinous |
| 82553 | Adenocarcinoma, mixed with other types of carcinoma incl. squamous cell and small-cell carcinoma |
| 82563 | Minimally invasive adenocarcinoma, non-mucinous |
| 82573 | Minimally invasive adenocarcinoma, mucinous |
| 82603 | Papillary adenocarcinoma, NOS |
| 82653 | Micropapillary adenocarcinoma |
| 83103 | Clear cell adenocarcinoma |
| 83333 | Fetal adenocarcinoma |
| 84703 | Mucinous cystadenocarcinoma |
| 84803 | Mucinous adenocarcinoma |
| 84903 | Signet ring cell carcinoma |
| 85503 | Acinar cell carcinoma |
| 85513 | Acinar adenocarcinoma |
|  |  |
|  | **Squamous cell carcinoma** |
| 80523 | Papillary squamous cell carcinoma |
| 80702 | Squamous cell carcinoma in situ |
| 80703 | Squamous cell carcinoma |
| 80713 | Keratinizing squamous cell carcinoma |
| 80723 | Non-keratinizing squamous cell carcinoma |
| 80733 | Squamous cell carcinoma, small cell non-keratinizing |
| 80833 | Basaloid squamous cell carcinoma |
| 80843 | Squamous cell carcinoma, clear cell type |
|  |  |
|  | **NSCLC NOS** |
| 80103 | Carcinoma, NOS |
| 80203 | Carcinoma, undifferentiated NOS |
| 80213 | Carcinoma, anaplastic NOS |
| 80463 | Carcinoma, non-small cell unspecified |
|  |  |
|  | **Large cell carcinoma (non-squamous NSCLC)** |
| 80123 | Large-cell carcinoma, unspecified |
|  |  |
|  | **Neuroendocrine NSCLC carcinoma (other specified NSCLC carcinoma)** |
| 80133 | Large cell neuroendocrine carcinoma |
| 82463 | Neuroendocrine carcinoma, NOS |
|  |  |
|  | **Other miscellaneous NSCLC (other specified NSCLC carcinoma)** |
| 80143 | Large cell carcinoma with rhabdoid phenotype |
| 80223 | Sarcomatoid carcinoma, pleomorphic |
| 80233 | NUT carcinoma |
| 80303 | Spindle cell and giant cell carcinoma |
| 80313 | Giant cell carcinoma |
| 80323 | Spindle cell carcinoma, NOS |
| 80333 | Pseudosarcomatous carcinoma |
| 81233 | Basaloid carcinoma |
| 82003 | Adenocystic carcinoma |
| 84303 | Mucoepidermoid carcinoma |
| 85603 | Adenosquamous carcinoma |
| 85623 | Epithelial-myoepithelial carcinoma |
| 89723 | Blastoma, pulmonary (pneumoblastoma) |
| 89803 | Carcinosarcoma, NOS |
| 89823 | Myoepithelial carcinoma |

ICD-10, International Classification of Diseases for Oncology, 10th Revision; NOS, not otherwise specified; NSCLC, non-small cell lung cancer; UNS, unspecified.

**Supplementary Table 2. Demographic and Clinical Characteristics of Total Population at Baseline According to Year of Diagnosis (Sweden)**

|  | **Sweden – All Incident NSCLC** | | | | | | | | | | |
| --- | --- | --- | --- | --- | --- | --- | --- | --- | --- | --- | --- |
|  | **2005**  **(N = 2482)** | **2006**  **(N = 2510)** | **2007**  **(N = 2605)** | **2008**  **(N = 2637)** | **2009**  **(N = 2722)** | **2010**  **(N = 2778)** | **2011**  **(N = 2864)** | **2012**  **(N = 2832)** | **2013**  **(N = 2827)** | **2014**  **(N = 2876)** | **2015**  **(N = 2934)** |
| **Age at NSCLC diagnosis, y**  Mean (SD)  Median (Q1–Q3)  Range | 68.6 (10.3)  69.0 (61.0-76.0)  23.0-97.0 | 68.9 (9.9)  69.0 (62.0-76.0)  30.0-97.0 | 68.7 (9.9)  69.0 (62.0-76.0)  25.0-93.0 | 68.8 (10.1)  69.0 (62.0-76.0)  23.0-94.0 | 69.3 (9.7)  69.0 (63.0-76.0)  29.0-97.0 | 69.4 (9.6)  69.0 (63.0-76.0)  22.0-95.0 | 69.5 (9.7)  69.5 (64.0-76.0)  23.0-96.0 | 69.7 (9.5)  70.0 (64.0-76.0)  22.0-99.0 | 70.1 (9.4)  70.0 (65.0-77.0)  23.0-94.0 | 70.3 (9.3)  71.0 (65.0-77.0)  26.0-95.0 | 70.4 (9.1)  71.0 (65.0-77.0)  27.0-96.0 |
| **Sex, n (%)**  Male  Female | 1370 (55.2)  1112 (44.8) | 1345 (53.6)  1165 (46.4) | 1304 (50.1)  1301 (49.9) | 1343 (50.9)  1294 (49.1) | 1357 (49.9)  1365 (50.1) | 1450 (52.2)  1328 (47.8) | 1481 (51.7)  1383 (48.3) | 1423 (50.2)  1409 (49.8) | 1429 (50.5)  1398 (49.5) | 1393 (48.4)  1483 (51.6) | 1425 (48.6)  1509 (51.4) |
| **TNM classification at diagnosis, n (%)**  I  II  IIIA  IIIB  IV  Missing | 414 (16.7)  80 (3.2)  177 (7.1)  508 (20.5)  1050 (42.3)  253 (10.2) | 391 (15.6)  100 (4.0)  154 (6.1)  471 (18.8)  1094 (43.6)  300 (12.0) | 422 (16.2)  94 (3.6)  180 (6.9)  502 (19.3)  1127 (43.3)  280 (10.7) | 422 (16.0)  108 (4.1)  187 (7.1)  512 (19.4)  1250 (47.4)  158 (6.0) | 507 (18.6)  136 (5.0)  200 (7.3)  500 (18.4)  1230 (45.2)  149 (5.5) | 346 (12.5)  205 (7.4)  264 (9.5)  211 (7.6)  1229 (44.2)  523 (18.8) | 397 (13.9)  213 (7.4)  308 (10.8)  276 (9.6)  1539 (53.7)  131 (4.6) | 479 (16.9)  187 (6.6)  310 (10.9)  242 (8.5)  1519 (53.6)  95 (3.4) | 464 (16.4)  194 (6.9)  283 (10.0)  256 (9.1)  1525 (53.9)  105 (3.7) | 474 (16.5)  226 (7.9)  293 (10.2)  248 (8.6)  1491 (51.8)  144 (5.0) | 457 (15.6)  216 (7.4)  353 (12.0)  270 (9.2)  1490 (50.8)  148 (5.0) |
| **Histology, n (%)**  Non-squamous  Adenocarcinoma  Large cell carcinoma  Squamous cell carcinoma  NSCLC NOS, n (%)  NSCLC other, n (%) | 1286 (51.8)  1147 (46.2)  139 (5.6)  664 (26.8)  487 (19.6)  45 (1.8) | 1319 (52.5)  1215 (48.4)  104 (4.1)  623 (24.8)  510 (20.3)  58 (2.3) | 1411 (54.2)  1319 (50.6)  92 (3.5)  670 (25.7)  489 (18.8)  35 (1.3) | 1531 (58.1)  1450 (55.0)  81 (3.1)  629 (23.9)  426 (16.2)  51 (1.9) | 1599 (58.7)  1494 (54.9)  105 (3.9)  652 (24.0)  407 (15.0)  64 (2.4) | 1695 (61.0)  1600 (57.6)  95 (3.4)  636 (22.9)  373 (13.4)  74 (2.7) | 1791 (62.5)  1700 (59.4)  91 (3.2)  654 (22.8)  344 (12.0)  75 (2.6) | 1851 65.4)  1750 (61.8)  101 (3.6)  626 (22.1)  292 (10.3)  63 (2.2) | 1833 64.8)  1732 (61.3)  101 (3.6)  651 (23.0)  283 (10.0)  60 (2.1) | 1884 (65.5)  1803 (62.7)  81 (2.8)  657 (22.8)  261 (9.1)  74 (2.6) | 1957 (66.7)  1887 (64.3)  70 (2.4)  672 (22.9)  247 (8.4)  58 (2.0) |

NOS, not otherwise specified; NSCLC, non-small cell lung cancer; Q, quartile; SD, standard deviation; TNM, tumor, nodes, metastasis.

**Supplementary Table 3. Demographic and Clinical Characteristics of Total Population at Baseline According to Year of Diagnosis (Denmark)**

|  | **Denmark – All Incident NSCLC** | | | | | | | | | | |
| --- | --- | --- | --- | --- | --- | --- | --- | --- | --- | --- | --- |
|  | **2005**  **(N = 2496)** | **2006**  **(N = 2577)** | **2007**  **(N = 2770)** | **2008**  **(N = 2774)** | **2009**  **(N = 2770)** | **2010**  **(N = 3015)** | **2011**  **(N = 3011)** | **2012**  **(N = 3052)** | **2013**  **(N = 3109)** | **2014**  **(N = 3230)** | **2015**  **(N = 3135)** |
| **Age at NSCLC  diagnosis, y**  Mean (SD)  Median (Q1-Q3)  Range | 66.9 (10.2)  68.0 (60.0-75.0)  31.0-98.0 | 67.2 (10.0)  68.0 (60.0-75.0)  33.0-97.0 | 67.6 (10.1)  68.0 (61.0-75.0)  29.0-95.0 | 67.9 (10.1)  68.0 (61.0-76.0)  30.0-96.0 | 68.0 (9.7)  68.0 (62.0-75.0)  35.0-95.0 | 68.4 (10.0)  69.0 (62.0-76.0)  33.0-95.0 | 68.8 (9.8)  69.0 (63.0-76.0)  31.0-95.0 | 68.8 (9.9)  69.0 (62.0-76.0)  22.0-96.0 | 68.8 (9.9)  69.0 (62.0-76.0)  30.0-96.0 | 69.7 (9.6)  70.0 (64.0-77.0)  18.0-97.0 | 69.6 (9.5)  70.0 (64.0-76.0)  27.0-96.0 |
| **Sex, n (%)**  Male  Female | 1346 (53.9)  1150 (46.1) | 1389 (53.9)  1188 (46.1) | 1470 (53.1)  1300 (46.9) | 1455 (52.5)  1319 (47.5) | 1447 (52.2)  1323 (47.8) | 1550 (51.4)  1465 (48.6) | 1540 (51.1)  1471 (48.9) | 1576 (51.6)  1476 (48.4) | 1544 (49.7)  1565 (50.3) | 1689 (52.3)  1541 (47.7) | 1587 (50.6)  1548 (49.4) |
| **TNM classification at diagnosis, n (%)**  I  II  IIIA  IIIB  IV  Missing | 310 (12.4)  147 (5.9)  210 (8.4)  428 (17.1)  1215 (48.7)  186 (7.5) | 279 (10.8)  127 (4.9)  195 (7.6)  460 (17.9)  1331 (51.6)  185 (7.2) | 344 (12.4)  138 (5.0)  210 (7.6)  458 (16.5)  1413 (51.0)  207 (7.5) | 322 (11.6)  126 (4.5)  361 (13.0)  314 (11.3)  1505 (54.3)  146 (5.3) | 295 (10.6)  184 (6.6)  349 (12.6)  293 (10.6)  1515 (54.7)  134 (4.8) | 341 (11.3)  253 (8.4)  371 (12.3)  322 (10.7)  1637 (54.3)  91 (3.0) | 356 (11.8)  229 (7.6)  378 (12.6)  313 (10.4)  1598 (53.1)  137 (4.5) | 429 (14.1)  267 (8.7)  358 (11.7)  287 (9.4)  1581 (51.8)  130 (4.3) | 466 (15.0)  262 (8.4)  353 (11.4)  302 (9.7)  1568 (50.4)  158 (5.1) | 491 (15.2)  303 (9.4)  382 (11.8)  283 (8.8)  1616 (50.0)  155 (4.8) | 505 (16.1)  286 (9.1)  427 (13.6)  275 (8.8)  1507 (48.1)  135 (4.3) |
| **Histology, n (%)**  Non-squamous  Adenocarcinoma  Large cell carcinoma  Squamous cell carcinoma  NSCLC NOS  NSCLC other | 1129 (45.2)  1064 (42.6)  65 (2.6)  647 (25.9)  637 (25.5)  83 (3.3) | 1151 (44.7)  1068 (41.4)  83 (3.2)  741 (28.8)  567 (22.0)  118 (4.6) | 1376 (49.7)  1268 (45.8)  108 (3.9)  787 (28.4)  511 (18.4)  96 (3.5) | 1411 (50.9)  1320 (47.6)  91 (3.3)  738 (26.6)  512 (18.5)  113 (4.1) | 1428 (51.6)  1385 (50.0)  43 (1.6)  738 (26.6)  492 (17.8)  112 (4.0) | 1635 (54.2)  1607 (53.3)  28 (0.9)  775 (25.7)  461 (15.3)  144 (4.8) | 1709 (56.8)  1686 (56.0)  23 (0.8)  782 (26.0)  366 (12.2)  154 (5.1) | 1848 (60.6)  1841 (60.3)  7 (0.2)  777 (25.5)  300 (9.8)  127 (4.2) | 1852 (59.6)  1842 (59.2)  10 (0.3)  823 (26.5)  272 (8.7)  162 (5.2) | 1949 (60.3)  1942 (60.1)  7 (0.2)  833 (25.8)  287 (8.9)  161 (5.0) | 1898 (60.5)  1894 (60.4)  4 (0.1)  816 (26.0)  270 (8.6)  151 (4.8) |

NOS, not otherwise specified; NSCLC, non-small cell lung cancer; Q, quartile; SD, standard deviation; TNM, tumor, node, metastasis.

**Supplementary Table 4. Demographic and clinical characteristics of the incident NSCLC population by stage in Sweden**

|  | **Incident stage I-IIIA NSCLC** | | | **Incident stage IIIB-IV NSCLC** | | |
| --- | --- | --- | --- | --- | --- | --- |
|  | **All** | **NSQ** | **SQ** | **All** | **NSQ** | **SQ** |
|  | **(N = 9241)** | **(N = 5386)** | **(N = 2822)** | **(N = 18,540)** | **(N = 11,461)** | **(N = 3752)** |
| **Age at NSCLC diagnosis (years)**  Median (Q1–Q3) | 70 (64–76) | 69 (63–75) | 71 (65–77) | 69 (63–76) | 69 (62–76) | 71 (65–77) |
| **Sex**  Male, n (%) | 4533 (49.1) | 2280 (42.3) | 1714 (60.7) | 9579 (51.7) | 5391 (47.0) | 2393 (63.8) |
| **Comorbidities^a^**  Chronic pulmonary disease, n (%)  Congestive heart failure, n (%) | 2110 (22.8)  806 (8.7) | 1086 (20.2)  414 (7.7) | 770 (27.3)  291 (10.3) | 3116 (16.8)  1437 (7.8) | 1664 (14.5)  806 (7.0) | 866 (23.1)  362 (9.6) |
| **TNM classification at diagnosis**  I, n (%)  II, n (%)  IIIA, n (%)  IIIB, n (%)  IV, n (%)  Missing, n (%) | 4773 (51.7)  1759 (19.0)  2709 (29.3)  0  0  0 | 3170 (58.9)  881 (16.4)  1335 (24.8)  0  0  0 | 1232 (43.7)  664 (23.5)  926 (32.8)  0  0  0 | 0  0  0  3996 (21.6)  14,544 (78.4)  0 | 0  0  0  2008 (17.5)  9453 (82.5)  0 | 0  0  0  1263 (33.7)  2489 (66.3)  0 |
| **Histology**  Non-squamous cell carcinoma, n (%)  Adenocarcinoma, n (%)  Large cell carcinoma, n (%)  Squamous-cell carcinoma, n (%)  NSCLC NOS, n (%)  Other miscellaneous NSCLC, n (%) | 5386 (58.3)  5094 (55.1)  292 (3.2)  2822 (30.5)  805 (8.7)  228 (2.5) | 5386 (100)  5094 (94.6)  292 (5.4)  0  0  0 | 0  0  0  2822 (100.0)  0  0 | 11,461 (61.8)  10,779 (58.1)  682 (3.7)  3752 (20.2)  2955 (15.9)  372 (2.0) | 11,461 (100.0)  10,779 (94.0)  682 (6.0)  0  0  0 | 0  0  0  3752 (100.0)  0  0 |

^a^In the Swedish registry, the full lookback period was available. In the Danish registry, the lookback period was 2 years.

NSCLC, non-small cell lung cancer; NOS, not otherwise specified; Q, quartile; SD, standard deviation; TNM, tumor, nodes, metastasis.

**Supplementary Table 5. Demographic and clinical characteristics of the incident NSCLC population by stage in Denmark**

|  | **Incident stage I-IIIA NSCLC** | | | **Incident stage IIIB-IV NSCLC** | | |
| --- | --- | --- | --- | --- | --- | --- |
|  | **All** | **NSQ** | **SQ** | **All** | **NSQ** | **SQ** |
|  | **(N = 10,054)** | **(N = 5280)** | **(N = 3548)** | **(N = 20,221)** | **(N = 11,255)** | **(N = 4439)** |
| **Age at NSCLC diagnosis, years**  Median (Q1-Q3) | 69 (63-76) | 68 (61-74) | 72 (65-77) | 68 (61-75) | 67 (60-74) | 71 (64-77) |
| **Sex**  Male, n (%) | 5208 (51.8) | 2244 (42.5) | 2315 (65.2) | 10,511 (52.0) | 5159 (45.8) | 2906 (65.5) |
| **Comorbidities, n (%)^a^**  Chronic pulmonary disease  Congestive heart failure | 1623 (16.1)  390 (3.9) | 720 (13.6)  175 (3.3) | 654 (18.4)  163 (4.6) | 2495 (12.3)  628 (3.1) | 1205 (10.7)  314 (2.8) | 672 (15.1)  168 (3.8) |
| **TNM classification at diagnosis, n (%)**  I  II  IIIA  IIIB  IV  Missing | 4138 (41.2)  2322 (23.1)  3594 (35.7)  0  0  0 | 2491 (47.2)  1117 (21.2)  1672 (31.7)  0  0  0 | 1206 (34.0)  935 (26.4)  1407 (39.7)  0  0  0 | 0  0  0  3735 (18.5)  16,486 (81.5)  0 | 0  0  0  1625 (14.4)  9630 (85.6)  0 | 0  0  0  1371 (30.9)  3068 (69.1)  0 |
| **Histology, n (%)**  Non-squamous cell carcinoma  Adenocarcinoma  Large cell carcinoma  Squamous cell carcinoma  NSCLC NOS  Other miscellaneous NSCLC | 5280 (52.5)  5188 (51.6)  92 (0.9)  3548 (35.3)  795 (7.9)  431 (4.3) | 5280 (100)  5188 (98.3)  92 (1.7)  0  0  0 | 0  0  0  3548 (100)  0  0 | 11,255 (55.7)  10,913 (54.0)  342 (1.7)  4439 (22.0)  3606 (17.8)  921 (4.6) | 11,255 (100.0)  10,913 (97.0)  342 (3.0)  0  0  0 | 0  0  0  4439 (100.0)  0  0 |

^a^In the Swedish registry, the full lookback period was available. In the Danish registry, the lookback period was 2 years.
NSCLC, non-small cell lung cancer; NOS, not otherwise specified; Q, quartile; TNM, tumor, nodes, metastasis.
